# A Health Promoting School Intervention in 10th Grade (“My Life – I Decide”): Protocol for a Pragmatic Controlled Trial

**DOI:** 10.2196/100471

**Published:** 2026-07-17

**Authors:** Nanna Wurr Stjernqvist, Anne Timm, Anders Blædel Hansen, Mads Bølling, Tilde Skjærlund Jensen, Helle Stuart, Charlotte Demant Klinker

**Affiliations:** 1 Copenhagen University Hospital – Steno Diabetes Center Copenhagen, Borgmester Ib Juuls Vej 83, Herlev, 2730, Denmark, 45 30248612; 2 Faculty of Medicine & Health, School of Health Sciences, UNSW Medicine, University of New South Wales, Sydney, New South Wales, Australia; 3 Center for Clinical Research and Prevention, Frederiksberg, Capital Region, Denmark; 4 Research Centre for Didactics and Pedagogy, VIA University College, Aarhus, Central Denmark Region, Denmark; 5 Centre for Health, Family and Leisure, Vallensbæk Municipality, Vallensbæk, Vallensbæk Strand, Denmark; 6 Health Promotion and Interventions, National Institute of Public Health, University of Southern Denmark, Copenhagen, Denmark

**Keywords:** health promoting schools, whole school approach, adolescents, mental health, health literacy, school well-being, system level impact, process evaluation

## Abstract

**Background:**

Health promoting school (HPS) interventions have the potential to improve adolescent health and well-being, but evidence regarding implementation and system-level impact in real-world school settings remains limited. “My Life – I Decide” (My Life) is a systems-oriented HPS intervention developed to strengthen positive mental and physical health, school well-being, and health-promoting school practices among 10th-grade students in Denmark.

**Objective:**

This study aims to describe the intervention, study design, and evaluation framework of the “My Life” intervention and its pragmatic controlled trial, including effectiveness, process, and system-level evaluations.

**Methods:**

The intervention is informed by the World Health Organization HPS framework and combines curriculum-based health education, action-oriented teaching, life-psychological approaches, and education outside the classroom. The intervention includes four phases: (1) preparation through cross-sectoral collaboration between health and education sectors; (2) planning and local adaptation; (3) delivery of a health education program; and (4) anchoring of health-promoting practices at the school level. Effectiveness is evaluated using a pragmatic controlled waiting-list trial design. Four intervention schools (11 classes) were matched with 4 control schools (15 classes), including approximately 416 students aged 15‐17 years. Primary outcomes include social and emotional competences, self-efficacy, mental well-being, health literacy, school connectedness, and student interpersonal relations. Student survey data are collected at baseline, postintervention, and follow-up, and effectiveness will be analyzed using multilevel mixed models. System-level impacts are assessed using a mixed-methods design, including school staff surveys, interviews with school and municipal stakeholders, and student focus groups. A realist-informed multimethod process evaluation examines implementation fidelity, acceptability, contextual factors, and mechanisms of impact across intervention schools. Data sources include observations, interviews, student registration, and postsession surveys completed by health consultants after each teaching session.

**Results:**

The study will generate quantitative and qualitative data on student outcomes, implementation processes, intersectoral collaboration, and development of health-promoting practices within schools. Findings from the effectiveness, process, and system-level evaluations will be triangulated to test and refine the initial program theory of the intervention. Recruitment of schools and student enrollment have been completed, and baseline data collection commenced in September 2025. Follow-up assessments are being conducted according to the study timeline. Qualitative data collection for the process and system-level evaluations were completed in June 2026.

**Conclusions:**

The “My Life” study will contribute knowledge on the implementation and evaluation of complex HPS interventions in real-world educational settings. The findings may inform future HPS initiatives and provide methodological insights into combining effectiveness, process, and system-level evaluations in adolescent health promotion research.

## Introduction

### Social Inequality in Adolescent Health and Well-Being

Adolescence is a key period for shaping lifelong health behaviors and positive development [1,2]. However, this stage is often characterized by declining well-being [3,4] and increasing health risk behaviors [5,6]. These patterns are strongly socially patterned, as adolescents’ health and well-being are significantly influenced by their parents’ socioeconomic status (SES) (eg, income, education, occupation, and social position) [7,8]. Adolescents from families with low SES face a higher risk of mental health challenges [9,10], engage more often in health risk behaviors [7,11], and often have fewer health assets [12,13] than adolescents from families with high SES [14]. Such health-related disadvantages are especially problematic during the transition from compulsory schooling to higher education or the labor market, increasing vulnerability and the risk of school dropout [15,16].

In Denmark (DK), one example of adolescents in the transitional educational phase is students attending the optional 1-year municipal 10th grade. Students in municipal 10th grade are more likely to come from families with low SES compared with peers who transition directly to upper secondary education [14]. They also report lower well-being and more challenges related to educational engagement and readiness [17], thereby increasing the risk of school dropout [18]. While empirical evidence about this group’s health behaviors remains limited, broader social inequalities are expected to affect their health and well-being, underscoring the need for health promotion interventions in municipal 10th-grade school settings.

### School Health Promotion

Schools engage all students regardless of SES or ethnicity, making them key settings for health promotion and for reducing health inequity [19-21]. Evidence suggests that school-based interventions addressing system-level changes are particularly important for supporting students from families with low SES [22]. The most prominent is the systems-oriented World Health Organization Health Promoting School (HPS) framework [23]. This extends beyond health education to include structural changes in schools’ physical and social environments and collaboration with parents and/or local communities. The HPS adopts a whole-school approach with three core components: (1) formal health education, (2) a supportive school ethos and environment, and (3) engagement with families and/or the wider community [24]. Studies show small but significant positive outcomes of HPS interventions, including modest improvements in physical activity, fruit and vegetable intake [25], mental well-being [26], social and emotional competences [27], and bullying [28]. Some studies also suggest that HPS approaches may be effective in promoting health literacy or health-related action competence [27]. Yet, evidence is limited on the effect of school-based health promotion approaches, such as the HPS, on adolescents at risk of poor mental health and health behavior due to socioeconomic factors [29].

### The HPS Approach and Didactic Methods

In Europe, the HPS approach emphasizes student participation and action-oriented teaching as forms of “Democratic health education” highlighting that health education is most effective when teaching is experiential, action-oriented, based on a holistic health concept, and participatory [30]. Evidence shows that such approaches within HPS can promote social and emotional competences [31] and health-related action competence [32], while similar student-centered and active learning approaches are associated with improved mental health and well-being among adolescents in secondary education [33]. Several didactic methods build on these principles, including the life-psychological method and education outside the classroom (EOtC). The life-psychological method emphasizes participation and actions through hands-on exercises and reflections and has been identified as a potential HPS method to promote well-being with promising results among youth in educational settings [34,35]. EOtC relocates parts of curriculum teaching to settings outside the school—nature and culture environments and societal institutions—linking classroom content to real-world learning environments. EOtC conducted in natural environments is highlighted as particularly effective in supporting student health and well-being outcomes [36]. EOtC more generally has been found to support school motivation [37], physical activity [38], and school-well-being [39], and EOtC aligns with HPS by emphasizing local community involvement and participation [40]. While these methods show promise for promoting health for young schoolchildren, their effectiveness in secondary education—particularly among adolescents from lower SES families—remains scarce. Furthermore, little is known about mechanisms that influence the outcomes in these groups. Altogether, the lack of evidence limits our understanding of how HPS interventions can address social inequalities in adolescent health.

### Implementing the HPS Approach

Despite its potential, the implementation of HPS approaches is often challenged by the complexity of school systems and constrained by implementation barriers such as competing academic demands, limited time and resources, and insufficient organizational support [41,42]. These challenges often lead to partial implementation of interventions [43], thereby limiting their effectiveness and sustainability [44,45]. This underscores the importance of supportive and context-sensitive implementation strategies in the field. Strengthening intersectoral collaboration between the health and education sectors at the municipal level may represent a relevant strategy to support the implementation of HPS interventions. In the Danish context, this is particularly relevant, as municipalities are legally responsible for prevention and health promotion, including school health promotion and education. However, health consultants are often disconnected from the school health education curriculum, and teachers frequently lack the necessary competence in this field [46]. A potential solution to secure the necessary human and financial resources is to strengthen the role of municipal health consultants in supporting implementation. Combined with “add-in” approaches that integrate health promotion into existing curricula and align with the core tasks of the school, such strategies may support more successful implementation of HPS interventions [46,47].

To promote health and well-being among Danish students in a transitional educational phase and overcome known implementation barriers, the HPS intervention “My Life – I Decide (My Life)” was developed. “My Life” is expected to promote positive mental and physical health and school well-being among 10th-grade students (aged 15‐17 y) and strengthen health promotion capacity in schools and intersectoral collaboration. The intervention seeks to achieve these aims through 4 intervention phases, operating at both the student and system levels. The aim of this study protocol is to describe the “My Life” intervention and the design of the pragmatic controlled trial evaluating its effectiveness, implementation processes, and system-level impacts in Danish 10th-grade schools. Specifically, the study evaluates: (study 1) effectiveness on student health and school well-being outcomes; (study 2) impacts on school health promotion capacity and intersectoral collaboration; and (study 3) how implementation mechanisms and contextual conditions influence outcomes across school settings.

## Methods

### The “My Life – I Decide” Intervention

**Figure 1.**
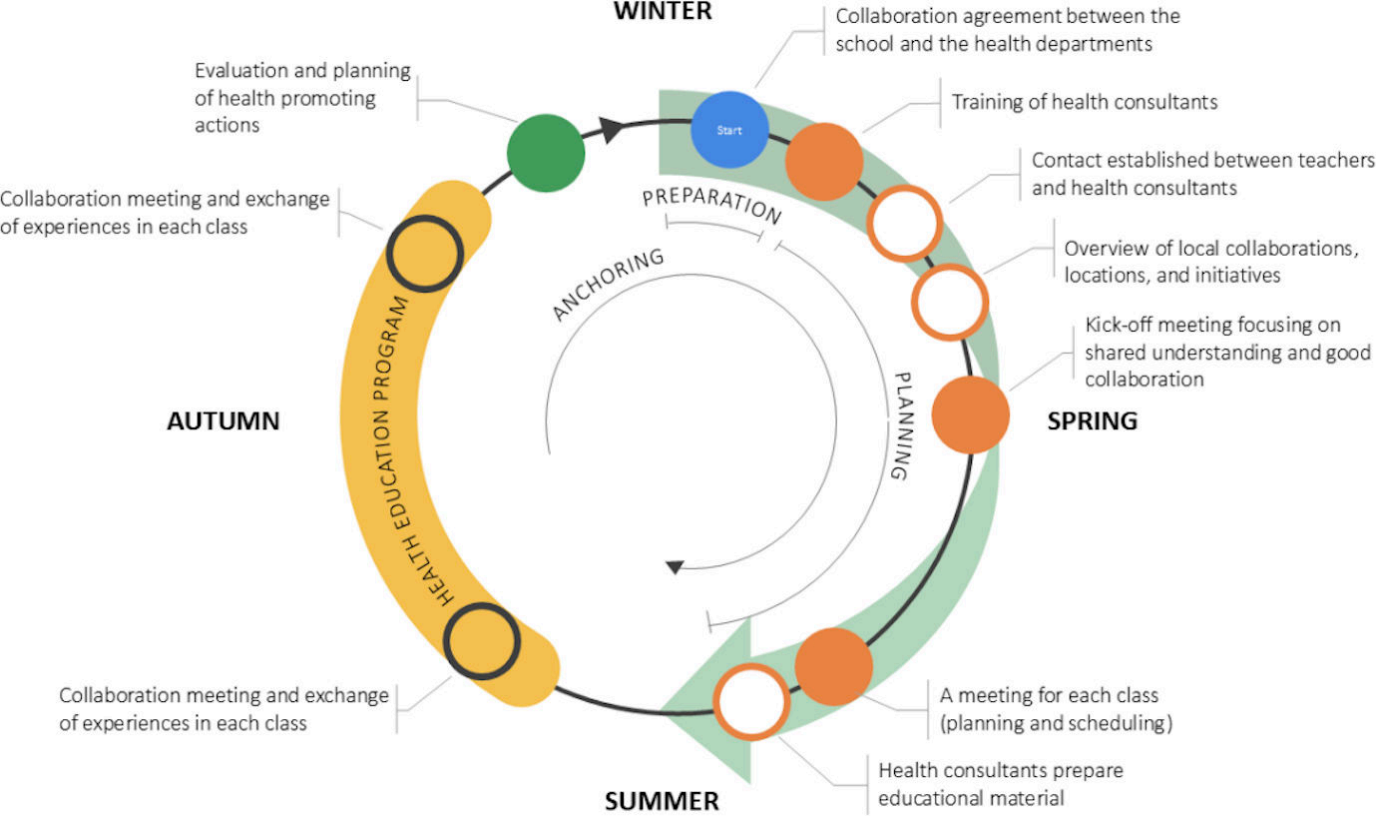
The “My Life” intervention. Activities are divided into four phases: (1) preparation (blue), (2) planning (orange), (3) health education program (yellow), and (4) anchoring (green). Filled circles indicate meetings or teaching sessions. An empty circle indicates an activity, such as making contact or preparing. The green arrow indicates ongoing anchoring and further development of health-promoting school practices.

The intervention is theoretically inspired by the HPS approach [24], action-oriented teaching [48], the life-psychological method [49], and EOtC [36]. Following common HPS definitions [25], “My Life” uses a whole school approach and operationalizes the 3 HPS components through 4 intervention phases. The phases are: (1) preparation: establishing collaboration between the school and municipal health department and building implementation capacity; (2) planning: adapting the program to schools; (3) health education program: classroom and community-based activities; and (4) anchoring: sustaining health-promoting work at the school. These phases are described in detail below and illustrated in Figure 1 (see also the Template for Intervention Description and Replication [TIDieR] checklist in Checklist 1). Municipal health consultants deliver the intervention in collaboration with school teachers and local actors, aligning with national priorities to strengthen collaboration between the health and educational sectors [46]. The intervention adapts to the school context with preparation and planning taking place before the summer break and school start, while the health education program and anchoring are implemented after the summer break through the full school year. The intervention serves as a framework for HPS practices that can be anchored at the school and further developed over time.

### Preparation

During the preparation phase, a formal collaboration agreement is established between the school and the municipal health department, outlining roles, responsibilities, political support, and resource commitments. To support implementation, a 2-day training course for health consultants is provided. This covers training in applied didactic methods and the application of the HPS framework. During the training, health consultants receive a comprehensive intervention manual developed to support program implementation. The manual is also provided to the schools for them to understand and be inspired by.

### Planning

This phase comprises three core activities that focus on organizing and tailoring the program to the local school context.

1. Establishment of a municipal peer-support network: The network enables health consultants from different municipalities to share experiences and receive backbone support from an experienced health consultant throughout the school year.
2. Start-up meeting between health consultants and 10th-grade teachers at the intervention schools: This meeting is conducted to build a shared understanding of roles and responsibilities during implementation and map already existing and potential collaborations between school and local community actors for the program to build on.
3. Planning meetings for each class: Meetings between teachers and health consultants for intervention school classes are held to tailor the implementation to local conditions.

### Health Education Program

The health education program is integrated into the 10th-grade curriculum as an “add-in” [47]. It comprises 15 sessions delivered at the class and community levels, over 11‐13 weeks early in the school year. The sessions cover 12 topics related to physical and positive mental health and school well-being (see Table 1). Most sessions occur in settings outside the school following the principle of EOtC. Health consultants facilitate the sessions, including supporting and motivating students and coordinating with community partners. Teachers participate and support students who need additional support. In the final session, health consultants collect students’ feedback from the program to be presented in the Anchoring phase. Teachers and health consultants are expected to continuously discuss how to integrate intervention methods into teaching practices to anchor the program within schools.

**Table 1.** Overview of health education program. The following overview provides an example of how the teaching program can be structured. The actual program is adapted and developed locally by each participating school and health consultant.

Sessions	Day	Time	Location	Activities
** 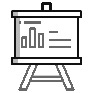 1. session: Intro meeting**	Tuesday	12:15 PM-2 PM	School	Overview of the “My Life” program, reflection on previous year’s youth survey results, and a group-building activity to demonstrate relevance and motivate active participation.
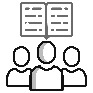 **2. session: A good life for me and the class rules**	Thursday	12:15 PM-2 PM	Shelter site	Team-building and class rule-setting to create a safe, inclusive, and positive classroom environment for the year.
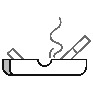 **3. session: Nicotine**	Tuesday	12:15 PM-2 PM	Nature school	Activities on nicotine’s effects, myth-busting, and support for quitting to increase knowledge, encourage reflection, and provide guidance on seeking help.
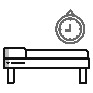 **4. session: Sleep**	Thursday	12:15 PM-2 PM	Shelter site	Activities that explore sleep habits, consequences of poor sleep, and strategies for improvement to support reflection on the link between sleep and well-being and provide tools for better sleep.
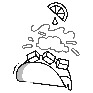 **5. session: Snack workshop**	Tuesday	12:15 PM-2 PM	In the school kitchen	Students prepare healthy snacks, explore their own habits, and learn how the Official Dietary Guidelines can be applied in simple, tasty ways.
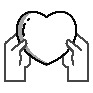 **6. session: Values**	Thursday	12:15 PM-2 PM	Shelter site	Students work with personal values and relate them to the class rules, gaining insight into themselves and the class community.
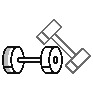 **7. session: Exercise**	Tuesday	12:15 PM-2 PM	Local fitness center	Students try out different types of exercise and explore what motivates them, gaining a positive experience with physical activity.
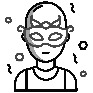 **8. session: My strengths and the strengths of others**	Thursday	12:15 PM-2 PM	Nature school	Students work with both body and inner strengths by guiding exercises and reflecting on personal qualities, building awareness of how these strengths support daily life and social settings.
** 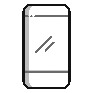 9. session: Social media**	Tuesday	12:15 PM-2 PM	Town hall	Students explore how social media affects behavior and well-being, using metaphors like ‘personal boundaries’ and ‘invisible communication’ to reflect on manipulation and miscommunication.
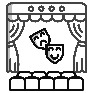 **10. session: Performance (part 1**)	Thursday	12:15 PM-2 PM	The local sports center	Through practical exercises, students strengthen focus, body awareness, communication, and cooperation, developing skills useful for exams, presentations, job interviews, and classroom well-being.
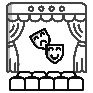 **11. session: Performance (part 2**)	Tuesday	12:15 PM-2 PM	The local sports center	Through practical exercises, students strengthen focus, body awareness, communication, and cooperation, developing skills useful for exams, presentations, job interviews, and classroom well-being.
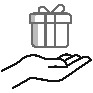 **12. session: Acts of kindness (part 1**)	Thursday	12:15 PM-2 PM	The local sports center or at the school gym	Students plan and carry out an activity by doing something active, together, and for others, allowing them to experience how meaningful shared actions can strengthen well-being and a sense of belonging.
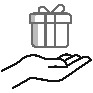 **13. session: Acts of kindness (part 2**)	Tuesday	12:15 PM-2 PM	The local sports center or at the school gym	Same as session 12: Acts of kindness (part 1)
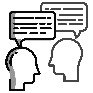 **14. session: Positive communities**	Thursday	12:15 PM-2 PM	Town hall	Students explore how nonverbal communication shapes group dynamics, define positive and negative communities, and plan a class event to reflect on the communities they want to be part of and how to contribute positively.
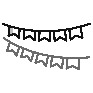 **15. session: Closing event**	Tuesday	12:15 PM-2 PM	Chosen by the students	The class completes the student-planned event, offering a shared experience and time to reflect on what the students have learned from the “My Life – My Choice” program.

### Anchoring

This phase comprises one main activity at school and community levels. An anchoring meeting is held at each school after the health education program. This is facilitated by health consultants who invite teachers, school and municipal management, relevant community actors, and students. Student feedback and experiences from the health education program are presented. This is expected to initiate a collaborative process to develop health-promoting actions for implementation. These actions are intended to build HPS capacity in schools. Actions may be student-driven, based on themes or needs identified by students, or system-driven, initiated by the school or municipality, such as integrating health topics across subjects or introducing structural initiatives.

### Program Theory and Determining Outcomes

**Figure 2.**
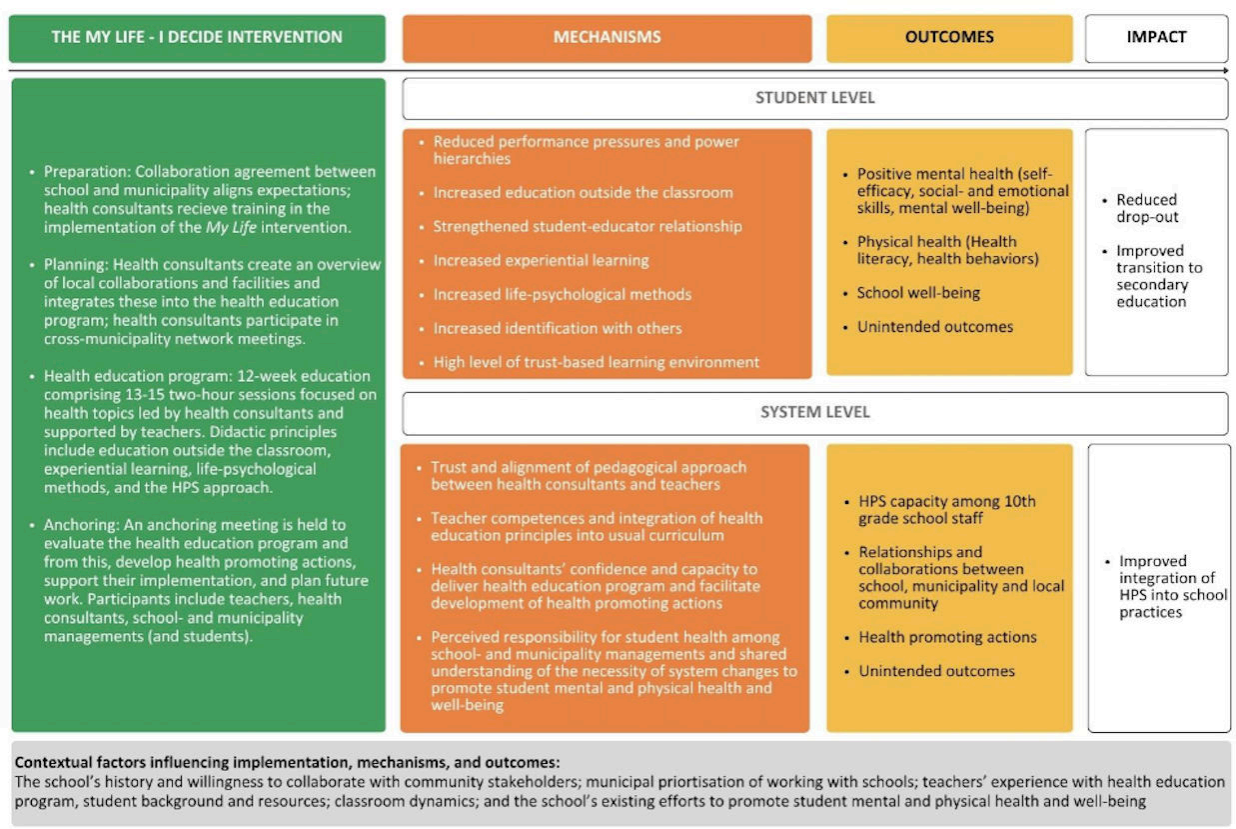
Program theory for “My Life – I Decide,” a health promoting school intervention. HPS: health promoting schools.

The intervention is underpinned by a program theory developed through a practice-research partnership between Vallensbæk Municipality, Steno Diabetes Center Copenhagen (SDCC), and the Intersectoral Prevention Laboratory [50]. The program theory outlines the final synthesis of the 4 main intervention phases, expected mechanisms of change, contextual factors, and expected outcomes at both the student and system levels. It illustrates how the intervention phases are expected to promote students’ physical and positive mental health and school well-being, health promotion capacity, and collaboration between school, municipality, and local community (Figure 2).

The development of the program theory guided the identification and selection of outcomes. Outcome selection was informed by both theoretical and empirical relevance, as well as stakeholder input, following recent recommendations for evaluating complex interventions [51]. Several primary and secondary outcomes were identified during the development of the intervention through qualitative data from students, health consultants, and 10th-grade teachers from Vallensbæk municipality, which was synthesized with existing evidence. At the student level, outcomes were grouped into three domains: positive mental health, physical health, and school well-being. Positive mental health was defined broadly to reflect students’ overall subjective well-being and psychological functioning [52], while school well-being refers specifically to students’ social relations and sense of belonging in the school. Physical health was addressed through health literacy as the primary outcome and several secondary indicators (health behaviors). This reflects the intervention’s focus on students’ competence and motivation, with health literacy understood as a key precursor for health-related behavior, whereas positive mental health and school well-being encompassed multiple subdomains. At the system level, outcomes were grouped under HPS practice and capacity, relationships and collaboration between the school, municipality, and local community, and health-promoting actions.

### Setting and Participants

The main settings are municipal 10th-grade schools and surrounding environments, including municipal health departments and various community organizations, such as fitness centers, scout groups, and town halls. Municipal 10th-grade students are the primary target group. In Denmark, compulsory schooling ends in the 9th grade. Students not yet prepared for upper secondary education have the option to enroll in 10th grade, a voluntary transitional year focused on academic and social growth with no admission requirements defining eligibility [1]. The 10th grade aims to boost students’ educational preparedness through academic enhancement, elective courses, and social and emotional development [2]. Hence, this context may suit the implementation of innovative health promotion interventions like “My Life.” Students attend daily from home without additional costs, unlike 10th-grade students at residents’ schools. The secondary target groups are school and municipal systems, where the intervention aims to strengthen health-promoting school practices and encourage collaboration.

### Study Design

**Figure 3.**
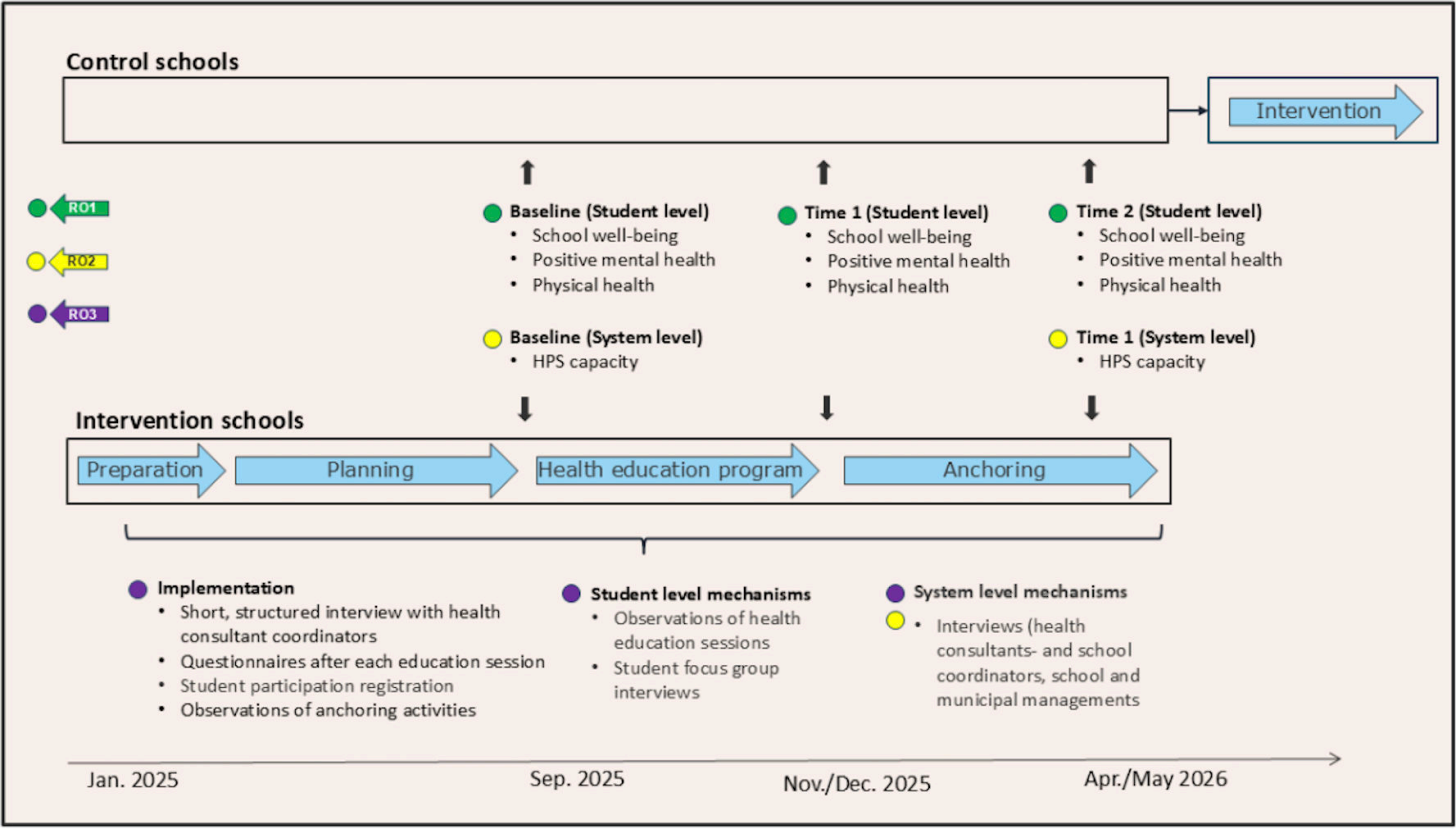
Study design of the “My Life” intervention. Green indicates data related to study 1, yellow indicates data related to study 2, and purple indicates data related to study 3. Student- and system-level data related to studies 1 and 2 are collected in both intervention and control schools, whereas data related to study 3 is collected in intervention schools only HPS: health promoting school.

Evaluating system-oriented HPS interventions such as “My Life” is challenging due to their multiple target groups, settings, and phases implemented at different organizational levels [51]. Consequently, the evaluation requires multiple context-adaptable designs and methods to address the 3 research objectives [51] (Figure 3). The different designs address the distinct research questions and are analytically complementary, capturing effectiveness, system-level impact, and implementation processes [53]. The evaluation encompassing all 3 research objectives will take place from January 2025 to June 2026. The effectiveness trial (study 1) follows a pragmatic controlled waiting list design with the participating schools as the assigned unit of allocation. Pragmatic trials are designed to determine intervention effects under real-world conditions rather than highly controlled efficacy trials [54]. The design and reporting of the trial are informed by the CONSORT (Consolidated Standards of Reporting Trials) extension for pragmatic trials. Schools are assigned to intervention either in 2025-2026 or 2026-2027. Schools scheduled to receive the intervention in 2026-2027 serve as controls in the trial. The system impact evaluation (study 2) and process evaluation (study 3) acknowledge that HPS interventions are context-bound and are approached through system thinking and realist evaluation. These approaches provide nuanced insights relevant for discussing potential scalability and for understanding outcomes and effects beyond the individual level (Bonell et al [55]: will it work here and for whom?). System-level impact (study 2) is evaluated through a mixed-method design inspired by Pinzon et al [56], while implementation processes at intervention schools (study 3) are examined using an adaptive multimethods design informed by realist evaluation [57], systems thinking [58], and guidance for developing and evaluating complex interventions [51]. The specific methods, data collection, and data analysis used to answer the 3 objectives are explained in detail below and illustrated in Table 2.

**Table 2.** Overview of data collection activities, timing, purpose, and analytical strategy.

Research objective, focus, and data collection activities	Timing	Purpose	Analytical strategy
1. Student-level outcomes			
Student surveys will be collected at baseline (T0; n≈416); approximately 2‐3 months later at mid-intervention (T1; n≈500), and approximately 4 months after that at follow-up (T2; n≈500)	August 2025-May 2026	To estimate effect changes in student mental and physical health and school well-being	Multilevel mixed models
2. System-level outcomes			
Health promoting school practice and capacity surveys collected among school staff at two time points (n≈70)	April-August 2025; May 2026	To describe differences in school staff health promotion (HP) practices	Pre-post test
Semi-structured interviews with school and municipality coordinators, and school and municipal managers (n=16)	April-June 2026	To identify systems impacts and map system activities, unanticipated outcomes and trace causal pathways across the school systems	The water of systems change [59]; Partnership Synergy Theory [60]
3. Implementation			
Short interviews with health consultant coordinators about the preparation and planning phases	August 2025	To assess implementation degree (high, medium, or low) for each intervention site	
Surveys administered after each health education session and anchoring meetings to health consultants	August-January 2025	To assess fidelity and acceptability of delivering “My Life”	Implementation outcomes [61,62]
3. Mechanisms			
Observations of health education sessions focusing on student engagement (n=20)	August-November 2025	To explore mechanisms of change at the student level	Realist-informed thematic analysis [63]
Semistructured FGIs^a^ with students across all four schools (n=4) and follow-up FGIs (n=4)	November-December 2025	To explore mechanisms of change at the student level	Realist-informed thematic analysis [63]
Semistructured interviews with school and municipality coordinators, and school and municipal managers (n=16)	May-June 2026	To explore mechanisms of change at the system level	CMO^b^-based deductive coding [59,60]

a FGI: focus group interview.

b CMO:context mechanism outcome.

### Matching and Assignment of Schools to Intervention and Control Schools

Before initiation of the “My Life” partnership, Vallensbæk municipality collaborated through the regional partnership “Cope with Life – Without Smoke and Drugs” [64] focused on adolescent health promotion. This partnership provided a practical framework for recruitment. Schools were recruited using convenience sampling, as participation was primarily limited to municipalities and 10th-grade schools already in the partnership. Through this partnership, 9 municipal 10th-grade schools were approached. Vallensbæk Municipality, which had implemented an earlier version of “My Life” for 5 years, served as the pilot school of the fully developed intervention. Of the remaining 8 schools, 7 agreed to participate following introductory meetings, while one declined due to a lack of interest. To balance intervention and control schools and account for anticipated student dropout, an additional control school from the region was recruited. During intervention development, randomization of schools was deemed unfeasible due to practical and organizational constraints, including partnership-based implementation context and differing readiness among schools to implement the intervention during 2025-26 or 2026-27. This is consistent with recommendations supporting nonrandomized designs when highly controlled conditions are not feasible [51]. Instead, schools were matched based on (1) their number of students and classes; (2) contextual and sociodemographic factors (including percentage of students with Danish ethnic backgrounds and average grades in mandatory exams); and (3) readiness to implement the intervention during the school years 2025-26 and 2026-27. Because schools were not randomized, baseline differences between intervention and control schools are expected. To assess potential selection bias, baseline student characteristics and school-level contextual characteristics will be examined descriptively across groups. Relevant baseline covariates, including gender, socioeconomic indicators, migration status, and school-level contextual factors, will be included in the mixed-effects analyses where appropriate. Four intervention schools with 11 classes were matched with 4 control schools with 15 classes. All enrolled 10th-grade students in participating classes were eligible to participate in the effectiveness trial, except students who could not read and understand Danish. All students were eligible to receive the intervention.

Because the study was implemented within an existing municipal partnership structure, the sample size was determined pragmatically by the number of participating schools eligible for implementation during the study period. Based on expected enrollment across the 8 schools, approximately 416 students are expected to participate. Although no formal a priori power calculation was performed during intervention development, sample size considerations were informed by comparable school-based quasi-experimental studies targeting adolescent health literacy and school well-being outcomes, which have demonstrated statistically significant intervention effects with similar sample sizes [65]. The study should therefore be considered adequately powered to detect small-to-moderate intervention effects at the student level, while acknowledging that statistical power for school-level effects may be limited due to the relatively small number of schools.

### Study 1: Effectiveness Evaluation at Student Level

#### Methods

The evaluation of student-level effectiveness will examine whether the intervention improves students’ positive mental and physical health and school well-being. This section describes the methods used to evaluate effectiveness within the pragmatic controlled trial design using repeated questionnaire measures collected at baseline and follow-up time points.

#### Primary Student-Level Outcomes

Positive mental health is operationalized through three aspects: social and emotional competences, self-efficacy, and mental well-being. These reflect both the hedonic perspective (mental well-being) and the eudaimonic perspective (social and emotional competences and self-efficacy) [66]. Social-emotional competence is measured using an index originally developed and tested for fifth to ninth-grade students [31], which covers three aspects—assertiveness, empathy, and collaborative skills. Self-efficacy is measured using a scale from the Danish Health Behavior in School Children (HBSC) which has been face-validated among students in grade 5‐9 [67]. Finally, mental well-being is measured using the validated shortened version of the Warwick-Edinburgh Mental Well-being Scale (WEMWBS) [68]. Physical health is measured using health literacy as both an indicator and determinant of health [69]. This is measured using the validated Danish version of the Health Literacy for School-aged Children (HLSAC) instrument, which covers five aspects: theoretical knowledge, practical knowledge, critical thinking, self-awareness, and citizenship [70]. School well-being is measured covering three aspects: positive student interpersonal relations, positive student-teacher relations, and school connectedness using items from the HBSC [71]. Student interpersonal relations are assessed using a student support scale, and student-teacher relations are assessed using a teacher-relatedness scale (ibid). Both scales have shown adequate validity and reliability in samples of 13- and 15-year-old students [72]. School connectedness is measured using a connectedness scale from the HBSC, which has shown good internal reliability in a subsample of Danish schoolchildren aged (11‐13 y) [45].

#### Secondary Student-Level Outcome

For secondary student outcomes, grouped under physical health, we will assess health behavior during school hours: (1) physical activity, which is measured using two items; (2) smoking and nicotine use, which is measured using 2 items; and (3) social media use, which is measured using one item. In addition, we will assess aspects of food literacy, using 3 adapted items of the validated Food Literacy Questionnaire [54].

#### Student Characteristics

At baseline, the following individual characteristics are collected: gender, age, migration status (with items on student and parental place of birth), and socioeconomic status (with items on parental employment and occupation), based on the standardized HBSC survey [11]. Moreover, family social structure is measured with 2 items [73], and language spoken mostly at home is measured with one item. Perceived social support is measured with one item on having someone to talk to when experiencing problems [74], and perceived school pressure is measured with one item on school-related pressure [11].

#### Data Collection

The indicators and research procedures were pilot tested during the school year 2024-25 through baseline and follow-up questionnaires and student focus group interviews (FGIs) (n=11) conducted in Vallensbaek municipality.

In the main trial, outcomes are measured through student surveys administered at 3 time points. Baseline data were collected in September 2025, approximately 4 weeks after the summer break. This timing was chosen based on findings from the pilot study, where students indicated that they needed more time in 10th grade to respond accurately to the questionnaires. A mid-intervention measurement (Time 1, T1) was collected immediately after the completion of the health education program (2 to 3 months after baseline). A follow-up measurement (Time 2, T2) will be collected approximately 7 to 8 months after baseline, allowing time to implement health promotion actions during the anchoring phase while remaining prior to the examination period (see Table 2, which outlines data collection for all 3 research objectives).

Data from intervention and control schools were collected concurrently. Student surveys were administered in schools during a 45-minute class session. Students were guided via video and instructed to wear headphones during the session. Researchers and trained interns supervised the class, supported technical issues, and prevented student interaction.

#### Analysis Plan

Scale validation will be conducted for the 3 constructs of positive mental health (self-efficacy, social and emotional competences, and mental well-being), 3 constructs of school well-being (positive student interpersonal relations, positive student-teacher relations, and school connectedness), and health literacy using Cronbach alpha to assess internal consistency. Intervention effects will be evaluated by mixed-effects modeling following the intention-to-treat principle. Data will be analyzed for each primary outcome separately, using models that account for the hierarchical school-based data structure, including repeated measurements within students and clustering of students within classes and schools. Random effects will be specified at relevant levels, where supported by the data, and the final random-effects structure will be selected based on the study design, data structure, model fit, and convergence. The effect will be estimated separately for each outcome with a group (intervention/control) × time interaction term, with time specified as a categorical variable with 3 levels (baseline, T1, and T2). The primary hypothesis for each outcome specified in the program theory concerns the comparison between the groups at T2, where the full intervention is implemented. All statistical tests will be 2-tailed, with the level of statistical significance set at *P*<.05. Each model will adjust for baseline values of the outcome and relevant confounders. Because the study is nonrandomized, baseline characteristics will be compared descriptively between intervention and control groups, and relevant baseline imbalances will be considered in the adjusted models where appropriate. Missing data will be described by group and time point. Mixed-effects models will use all available data under standard likelihood-based assumptions, and the potential impact of missing data will be considered when interpreting the findings. Model assumptions and graphs will be checked prior to analysis.

### Study 2: Systems Impact

#### Methods

The evaluation of system-level impact will investigate whether the intervention influences school, municipal, and community systems in ways that support the creation and maintenance of a HPS environment at the 4 intervention sites. This includes (1) measuring changes in school capacity for health promotion, (2) mapping health-promoting actions beyond the health education program, and (3) investigating cooperation and relationships between the municipal health departments, local communities, and schools, as well as identifying unexpected outcomes.

School health promotion capacity will be assessed using the validated Health Promoting School Practices and Capacity Questionnaire (HPS-Q) [75]. This survey consists of 24 items across seven subdimensions: (1) school policies, (2) school ethos, (3) collaboration and involvement, (4) school practice, (5) quality of delivery, (6) physical and financial resources, and (7) school health services. Items related to specific health topics will be adapted to the content of “My Life” following recommendations by Hjort et al [75]. The questionnaire will also include personal characteristics of respondents (gender, age, years working with the target group, years at the workplace, and teaching subjects). To investigate development in health-promoting actions, cooperation and relationships between the municipal health department, local community, and schools, and adverse positive and negative effects, semistructured interviews will be conducted with key actors across intervention schools (Table 2).

#### Data Collection

All school staff working with the involved 10th-grade students will be invited to complete an online survey in SurveyXact [76] to assess changes in HPS capacity. HPS-Q data will be collected at two time points: baseline (April-May 2025 including an additional round for newly employed staff in August 2025) and follow-up (April-May 2026). This period depicts the entire school year, after which students will transition to upper secondary education, employment, or other. System-level changes may first be detected after several years. However, small impacts are expected following the system-level processes integrated into the intervention. To detect these small changes, questions will be added in the HPS-Q survey to assess whether health promotion actions have been developed. The anchoring phase will be assessed; online surveys will be developed specifically for health consultant coordinators, and semistructured interviews will be conducted in April-June 2026 with school- and health consultant coordinators and school and municipal managers across intervention schools (n=16). The interview guides will include themes related to school-municipality collaboration, changes to school practice, unexpected system impacts, and health promotion actions developed as a result of the intervention.

#### Analysis Plan

Quantitative survey data will be analyzed descriptively at the school level across the 7 subdomains to capture changes in HPS capacity and practices over time. For each school, mean scores on the 7 subscales and the total HPS score will be calculated at baseline and at follow-up. Change scores will be computed as the mean difference between follow-up and baseline and summarized separately for intervention and control schools.

Qualitative analysis will begin with the transcription of the primary content from the audio recordings generated during the semistructured interviews. Partnership synergy theory [60] will be used as a deductive coding framework to analyze transcripts in relation to municipal-school collaborations. This will be merged with inductive coding, which will explore unexpected system impacts. The transcribed material related to the developed health-promoting actions will be compiled into an action registry in Microsoft Excel, collating data from all 4 intervention sites. This registry will contain: (1) detailed descriptions of each action implemented during the program period, (2) the process of their implementation, and (3) expected system impact. The action registry will be used to depict the relationships between actions and their impacts during the school year. Analysis will proceed in 3 steps. First, we will quantify the actions taken at each study site. Second, we will classify identified impacts according to the Six Core Dimensions of Systems Change [59] to provide a structured typology of system impact. Third, a cross-case comparison [77] will be conducted of the 4 intervention schools supported by site-level findings from the semistructured interviews to identify significant mechanisms across schools that contributed to systems impact [78].

### Study 3: Process Evaluation

#### Methods

The process evaluation will assess how the intervention was implemented across intervention school settings, including fidelity and acceptability, and what mechanisms were triggered at student and system levels. The evaluation will follow a realist-informed approach, integrating deductive, inductive, and theory-driven analyses to understand how context and mechanisms interact to produce student and system-level outcomes.

#### Data Collection

Implementation and mechanism data will be collected across the 4 intervention schools from January 2025 to June 2026, thus covering all 4 intervention phases of “My Life.” For implementation (1), the following data will be collected: short online structured interviews with health consultant coordinators about the Preparation and Planning phases to assess implementation degree, that is, fidelity guided by Proctor et al [61]. Postsession surveys with health consultants will be collected using SurveyXact [76] following each health education session across the eleven intervention classes (n=224) and the anchoring meeting (n=4) (September-January 2025) to capture fidelity and acceptability [61].

For student-level mechanisms (2), the following data will be collected: observations of health education sessions in 6 different classes across the 4 intervention schools (n=23) (August-November 2025) to investigate delivery context and student engagement. Student FGIs will be completed after the health education program in one of the observed classes at each school (n=4) (November-December 2025) to examine perceived relevance and engagement. FGIs will be repeated in the same classes (n=4) (April-May 2026), if HPS actions are implemented in schools to explore how these affect students.

For system-level mechanisms (3), the semistructured interviews described in the systems impact study (study 2) will be used to examine contextual barriers, enablers, and organizational factors influencing implementation and potential scalability (April-June 2026).

#### Analysis Plan

The process evaluation will draw on a combination of abductive and realist-informed analytical approaches. Data analysis will proceed in 3 parallel but interconnected strands, corresponding to the three objectives: (1) implementation, (2) student-level mechanisms, and (3) system-level mechanisms. Across all strands, we will integrate findings through a realist logic of analysis using the CMOs (context-mechanism-outcome configurations) produced through the intervention development and piloting phases (Timm et al [50]) to be tested, refined, or refuted through empirical data.

To assess implementation (1), implementation degree will be coded as either low, moderate, or high across each of the 4 intervention phases (preparation, planning, health education, and anchoring) based on the short structured online interviews with health consultants (September 2025-January 2026) and the follow-up interviews (May-June 2026), and postsession surveys among health consultants. Open-ended responses to postsession surveys will be coded against Proctor’s implementation outcomes (2011). The postsurveys developed for the anchoring meeting will be used to assess fidelity and to identify emerging system-level mechanisms influencing the integration of health promotion actions at the school, municipal, and community levels.

To assess student-level mechanisms (2), fieldnotes from observations of health education sessions will be subject to realist-informed thematic analysis to confirm, refine, or refute CMOs. FGIs will be coded deductively against these CMOs, with inductive coding applied to capture new or unexpected mechanisms.

To assess system-level mechanisms (3), semistructured interviews inspired by systems theory [59] with teachers, health consultants, and school and municipal managers will be analyzed using dimensional analysis to identify cross-cutting themes, barriers, and enablers across schools. Attention will be paid to the CMOs at the system level, for example, health consultant-teacher collaboration and management support identified in the developing and piloting phases. Surveys from the anchoring meetings and semi-structured interviews will contribute to the identification of system-level mechanisms supporting or constraining the implementation of health-promoting actions. This analytical process will be integrated with the program theory.

### Ethical Considerations

The “My Life” effectiveness trial was registered in ClinicalTrials.gov NCT07411677. The study was reviewed by the Research Ethics Committee for Science and Health in the Capital Region of Denmark, which determined that formal ethical approval was not required under Danish legislation regulating biomedical research (reference number F-25020538). This decision was based on the study involving questionnaire surveys, interviews, focus groups, and observations without collection of biological material or biomedical interventions. Written informed consent will be obtained from all participants prior to data collection, including students in surveys and FGI, and school and municipal staff in interviews and surveys. Under Danish regulations for nonbiomedical research, adolescents aged 15 years and older may independently provide consent for participation in questionnaire- and interview-based research [79]. Parents and guardians were informed about the study through school communication systems before data collection and had the opportunity to contact the school or research team with questions or concerns. Parents or guardians were informed via school communication, and parental consent was not required under national regulations for nonbiomedical research. Parents or guardians of participating students were informed about the study through school communication channels, and in accordance with national regulations for nonbiomedical research involving adolescents, parental consent was not required. Videos explaining “My Life” and the implications of giving consent were shown in classes before collecting student consent. Data will be collected, processed, and securely stored in accordance with rules and regulations of the Danish Data Protection Agency. The project follows the Danish Code of Conduct for Research Integrity and the Helsinki II Declaration principle.

## Results

**Figure 4.**
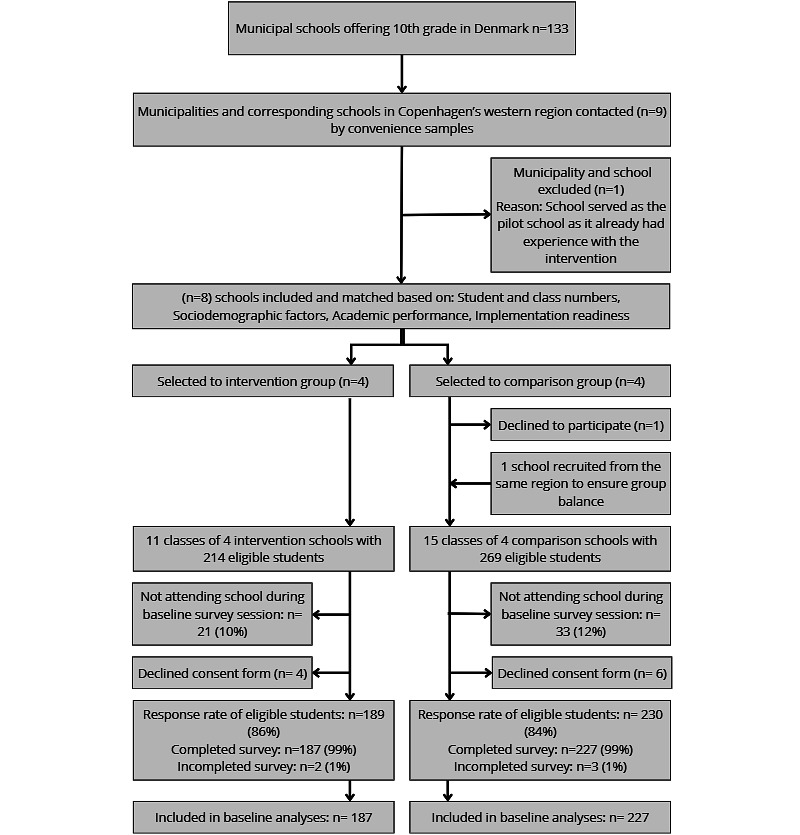
Flowchart of recruitment stages and participants included at baseline in the effectiveness trial.

At the time of submission, recruitment of schools and student enrollment have been completed. Baseline data collection commenced in September 2025. Follow-up assessments are being conducted according to the predefined study timeline. Qualitative data collection for the process and system-level evaluations will continue until June 2026. An overview of the recruitment process is shown in Figure 4.

## Discussion

### Expected Findings

The “My Life” study will provide new knowledge on the implementation and evaluation of a systems-oriented HPS intervention targeting older adolescents in Danish 10th-grade schools, including an updated program theory revealing key mechanisms of change. Adolescent mental health and school well-being are shaped by complex interactions between individual, social, educational, and organizational factors, and HPS approaches have been suggested as relevant strategies for addressing such complexity [25]. The intervention will be implemented in real-world school settings characterized by diverse student needs and varying organizational capacities and will provide critical insights relevant to similar educational settings.

### Strengths of the Study Design

The “My Life” intervention combines curriculum-based health education with broader HPS principles, including intersectoral collaboration and local anchoring processes. A major strength of the study is the integration of effectiveness, process, and system-level evaluations within a single evaluation framework. This enables examination of both student-level outcomes and the contextual and organizational processes influencing implementation.

Another strength is the pragmatic design, allowing the intervention to be implemented under real-world conditions in collaboration with schools and municipalities. The close collaboration between researchers, municipalities, and schools during intervention development and implementation may strengthen contextual relevance and future scalability. Furthermore, the extensive use of qualitative and quantitative methods across multiple stakeholder groups, including students, teachers, school managers, and municipal health consultants, may provide a broader understanding of how HPS interventions are implemented and adapted within educational systems.

### Potential Challenges

Potential challenges include variation in implementation across schools due to differences in organizational capacity, management support, competing educational priorities, and local adaptation of the intervention. While flexibility is consistent with HPS principles and pragmatic trial methodology, it may also influence implementation fidelity and comparability across schools.

In addition, sustaining health-promoting activities beyond the educational program itself may be challenging due to limited time and resources within schools and municipalities. Another challenge relates to the evaluation of systems-level change, as organizational and cultural changes within schools have been found to take more than one school year [28] and thus may extend beyond the study period. Finally, because the study uses a nonrandomized pragmatic design, there is a potential risk of selection bias compared with randomized controlled trials. However, the pragmatic approach was chosen to ensure feasibility and relevance within existing municipal and school structures.

### Conclusions

In conclusion, the “My Life” study may contribute important knowledge about how systems-oriented HPS interventions can be implemented and evaluated in transitional educational settings. The study may inform future development of school-based health promotion initiatives targeting older adolescents and strengthen understanding of how contextual and organizational conditions influence implementation processes and school-level change. Furthermore, the study may contribute methodological insights relevant for future evaluations of complex public health interventions implemented within real-world school systems.

## Supplementary material

10.2196/100471 Checklist 1 TIDieR checklist.

10.2196/100471 Peer Review Report 1 Peer Review Report by TrygFonden (Denmark).

## Abbreviations

CONSORT: Consolidated Standards of Reporting Trials
EOtC: education outside the classroom
FGI: focus group interview
HPS: health promoting school
HPS-Q: Health Promoting School Practices and Capacity Questionnaire
SDCC: Steno Diabetes Center Copenhagen
SES: socioeconomic status
WEMWBS: Warwick-Edinburgh Mental Well-being Scale
