# Supplementary material for: A Health Promoting School Intervention in 10th Grade (“My Life – I Decide”): Protocol for a Pragmatic Controlled Trial
Source: JMIR Res Protoc. 2026 Jul 17;15:e100471. doi: 10.2196/100471 (PMC13378738; doi:10.2196/100471)
Supplement: Checklist 1 [file resprot-v15-e100471-s001.docx]

**TIDieR checklist for the My Life – I Decide intervention**

**1. Brief name**

My Life

**2. Why (rationale, theory, goals)**

My Life is grounded in the WHO Health Promoting Schools (HPS) framework and principles of democratic health education. The intervention aims to promote positive mental and physical health and school well-being among students in Danish municipal 10th-grade schools by strengthening social and emotional competences, health literacy, self-efficacy, and health-related action competence.

The intervention is informed by a program theory developed through a practice–research partnership. At student level, the program theory posits that participatory, action-oriented, and experiential teaching—drawing on the life-psychological method and education outside the classroom (EOtC)—triggers mechanisms such as engagement, relevance, reflection, collective participation, and perceived agency. These mechanisms are expected to improve students’ positive mental health, physical health, and school well-being.

At system level, the program theory assumes that intersectoral collaboration between schools, municipal health departments, and local community actors—supported by municipal health consultants—triggers mechanisms related to shared ownership, capacity building, and alignment with core school tasks. These mechanisms are expected to strengthen schools’ health promotion capacity, collaborations, and sustainable health-promoting actions.


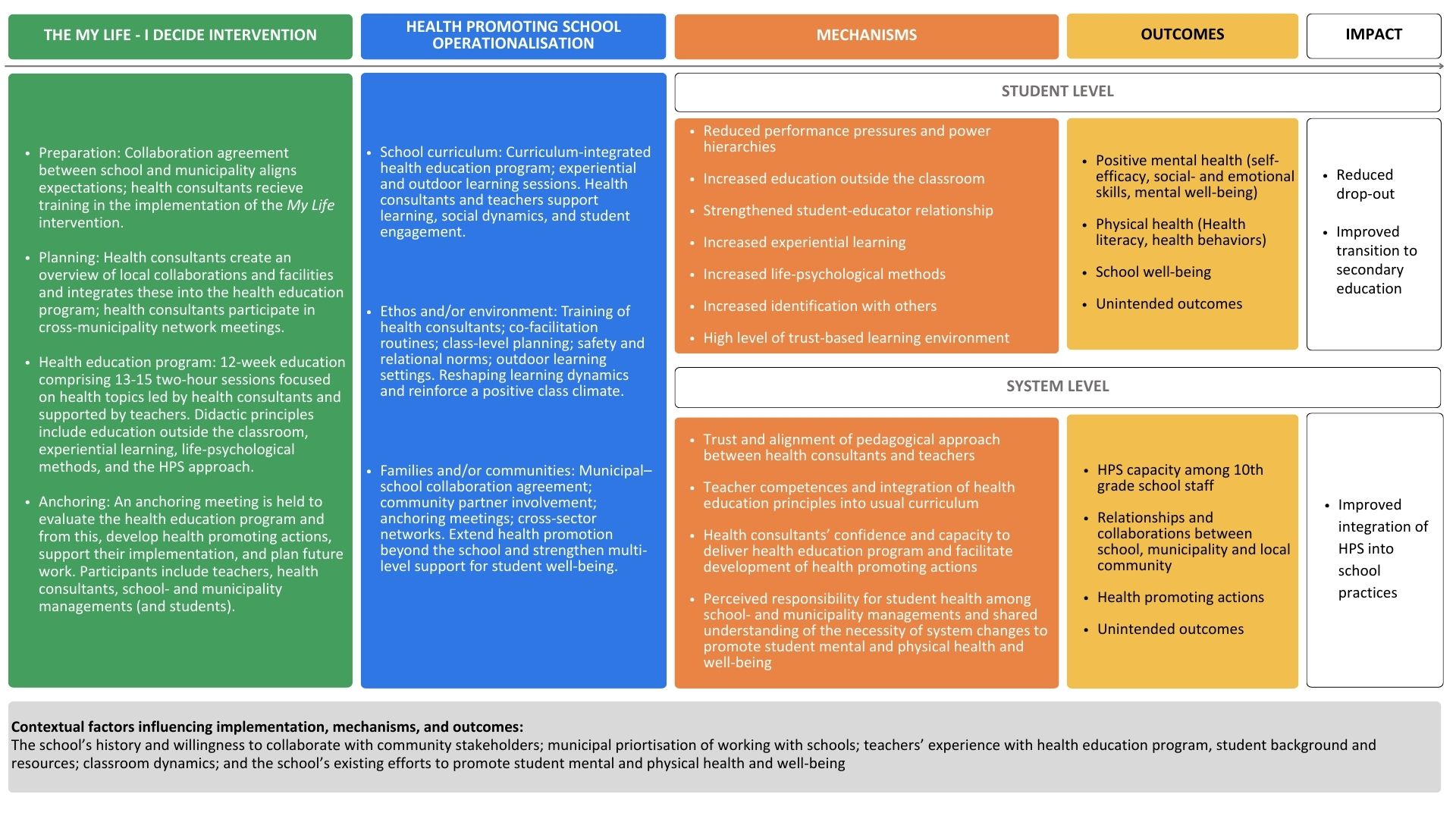


**3. What (materials)**

Implementation is supported by a comprehensive intervention manual for municipal health consultants and teachers. Materials include structured lesson plans, session outlines, reflection and dialogue exercises, student worksheets, and guidance for active student participation facilitation. Materials support both classroom-based activities and EOtC sessions conducted in natural and community-based environments.

**4. What (procedures)**

The My Life intervention is implemented through four sequential and interconnected phases operating at both student and system levels.

**Phase 1: Preparation**

The Preparation phase consists of establishing a collaboration agreement between schools and municipal health departments and educate health consultants to implement the intervention. First a formal collaboration agreement is established between the school and the municipal health department. This agreement outlines roles, responsibilities, political support, and resource commitments. To support the implementation municipal health consultants, participate in a two-day training program prior to implementation. Day one focuses on hands-on training in didactic methods, including action-oriented teaching, the life-psychological method, and EOtC. Day two focuses on applying the HPS framework to school practice, including planning of post-program anchoring activities. During the trial, the training was delivered by the non-governmental organization Hello Kitchen, which specializes in professional health promotion programs for practitioners. Health consultants receive a comprehensive intervention manual to support delivery and collaboration with schools.

**Phase 2: Planning**

The planning phase builds on three core activities, which focus on organizing and tailoring the program to the local school context. First, a municipal peer-support network is established. This enables health consultants from different municipalities to share experiences and receive backbone support from an experienced health consultant throughout the implementation period/school year is established. Second, health consultants and 10th-grade teachers participate in a start-up meeting to build a shared understanding and commitment to the collaboration and map existing and potential collaborations between school and local community actors. Third, planning meeting(s) between teachers and health consultants are held to tailor the implementation of the health education to local conditions including planing the dates and timetables for the health education sessions.

**Phase 3: Health education program**

The health education program constitutes the core student-level component of the intervention. It is delivered as an “add-in” to the existing 10th-grade curriculum and comprises 15 structured sessions of approximately 60 minutes delivered over 11–13 weeks early in the school year. The sessions cover 12 topics related to positive mental and physical health and student well-being e.g., A good life for me and Class rules, Nicotine, Sleep, and Performance. The program is grounded in a broad and positive health concept. The didactic principles include active student participation and experiential hands-on learning activities. Most sessions take place in outdoor settings e.g., local shelters, gym halls, or the town hall in line with the principles of EOtC.

**Phase 4: Anchoring**

After the health educational program an anchoring meeting, is held at each school. Health consultants facilitate anchoring meetings involving teachers, school management, municipal representatives, and, where relevant, students and local community actors. Student feedback and experiences from the program are used to collaboratively identify, develop, and plan health promoting actions to be embedded in everyday school practice. Actions may be student-driven, based on themes or needs or system-driven initiated by the school or municipality.

**5. Who provided**

The intervention is primarily delivered by trained municipal health consultants. Health consultants hold professional expertise in health promotion, act as a neutral facilitator for reflection and dialogue, and help build the school’s own health competencies so that the program can be sustainably anchored. School teachers participate in sessions, support students, and contribute to anchoring health-promoting practices. Local community actors may contribute to selected sessions.

**6. How**

The intervention is delivered face-to-face through group-based teaching sessions using participatory, dialogical, and experiential pedagogical methods. Delivery includes both in-school teaching and EOtC sessions conducted outside the school setting.

**7. Where**

The intervention takes place in municipal 10th-grade schools, including classrooms and school outdoor areas, as well as in local community settings such as natural environments, sports facilities, and municipal institutions.

**8. When and how much**

The health education program consists of 15 sessions of approximately 60 minutes delivered over 11–13 weeks early in the school year. Preparation and planning activities are conducted prior to the start of the school year, while anchoring activities take place following completion of the health education program and continue thereafter. The overall intervention spans approximately one and a half school years, with system-level activities extending beyond the health education program.

**9. Tailoring**

The intervention is tailored to local school contexts through planning meetings between health consultants and teachers. Tailoring includes selection of settings, involvement of community actors, and alignment with local priorities, while maintaining core components and mechanisms specified in the program theory.

**10. Modifications**

No predefined modifications to the intervention protocol are planned. Any adaptations occurring during implementation will be documented through surveys, observations, and interviews and analyzed as part of the process evaluation. These findings will inform future refinement of the intervention.

**11. How well (planned)**

The My Life intervention will be evaluated using a multimethod, pragmatic study design addressing effectiveness at student level, system-level impact, and implementation processes. Student-level outcomes related to positive mental and physical health and school well-being will be assessed using repeated questionnaire measures in intervention and control schools. System-level impacts will be evaluated through pre- and post-intervention staff surveys at intervention and control schools and qualitative interviews with school and municipal stakeholders. Implementation processes and mechanisms will be explored using a realist-informed, multi-method approach including observations, surveys, interviews, and focus groups with students, teachers, health consultants, and school and municipal representatives. Findings from the evaluation will inform refinement of the intervention and support future implementation and scaling

**12. How well (actual)**

Actual delivery, fidelity, and adaptations of the My Life intervention will be examined through the process evaluation using a realist-informed, multi-method design. Data sources include student participation logs and post-session surveys completed by health consultants, observations of health education sessions, short interviews with health consultants, focus group interviews with students, and interviews with teachers, health consultants, and school and municipal managers. These data will be used to document what was delivered in practice, variation across schools, and how contextual factors and mechanisms influenced implementation
