# Supplementary material for: A Health Promoting School Intervention in 10th Grade (“My Life – I Decide”): Protocol for a Pragmatic Controlled Trial
Source: JMIR Res Protoc. 2026 Jul 17;15:e100471. doi: 10.2196/100471 (PMC13378738; doi:10.2196/100471)
Supplement: Peer Review Report 1 [file resprot-v15-e100471-s002.pdf]

Reviewerne er de samme som ved bedømmelserne af ID 155926

Review 1

Applicant: Charlotte D Klinker File number: 158 775

		Excellent	Very good	Good	Fair	Poor	Do not know / NA
Quality of the project	- Basic idea		x				
	- Originality		x				
	- Methodology		x	x			
Qualifications of applicant			x				
Project feasibility			x				
Probability by which the likely outcomes of the project could improve practice in the field		x	x				
Probability by which the likely outcomes could contribute to the research in the field			x				
Plan for dissemination		x	x				
Overall assessment of the project		(x)	xx	(x)			

Comments

1. Quality of the project or project plan

I think the applicants did a nice job in responding to review comments. The application is improved and clearer in several scientific/methodological aspects. I went through the detailed responses to all reviews. From what I understand, these replies were sufficient and well thought out.

My main objection is the lack of scientific rigor in terms of informing the reader about sample sizes, design, analytic strategy/analysis, main outcomes, limitations and strengths of the studies cited.

However, the protocol has improved in several other aspects, and I guess the lack of numbers arise from the scientific tradition within prevention research, which is problematic.

2. Qualifications of the applicant

Improved, as it is the group leader who now applies.

3. Project feasibility

100 % documented that this can be done.

4. Probability by which the likely outcomes of the project could improve practice in the field

More difficult to evaluate, as there is no clear link btw. a scientific finding and the application in everyday management in this area.

5. Probability by which the likely outcomes could contribute to the research in the field

I am definitely positive towards this, and maybe even more positive if some quantitative aspects became part of the scientific reports.

6. Plan for dissemination

Quite detailed and well presented.

7. Overall assessment of the project

Improved. The existence of quite large pilot data showing positive effects further underlines my positive scoring of the application.

8. Other comments, including any proposals for improvement of the project

Review 2

Applicant: Charlotte D Klinker File number: 158 775

		Excellent	Very good	Good	Fair	Poor	Do not know / NA
Quality of the project	- Basic idea	x					
	- Originality			x			
	- Methodology		x				
Qualifications of applicant			x				
Project feasibility		x					
Probability by which the likely outcomes of the project could improve practice in the field			x				
Probability by which the likely outcomes could contribute to the research in the field		x					
Plan for dissemination				x			
Overall assessment of the project			x				

Comments

Thank you for drafting these detailed responses to the previous reviewer comments. I appreciate the effort put forth by the research team in designing this program and would like to provide feedback on various aspects of the project.

It is great to see that the feedback has been taken onboard and integrated into the revised plans of the development and feasibility trial of the school based 'My Life – I decide' mental and physical activity literacy program. Below are my point-by-point responses for the 8 quality assessment criteria.

1. Quality of the project or project plan

The project plan for the 'My Life – I decide' development and evaluation study demonstrate a clear understanding of the importance of mental and physical activity literacy among secondary-school aged children. The goals and objectives of the 'My Life - I Decide' program are well-defined and align with the aim of promoting holistic well-being and youth autonomy.

The plan incorporates relevant theoretical frameworks (e.g. PRECIS, CONSORT Extension for Pragmatic Trial checklist) and evidence-based approaches, which enhances the overall quality of the project. The structure and organization of the project plan are commendable, with logical sequencing of activities, a comprehensive evaluation strategy and feasible (if tight) timeline outlined.

2. Qualifications of the applicant

The qualifications of the team of applicants indicate a strong background in public health research and expertise in the field of mental and physical activity, complex intervention research and familiarity with co-production of interventions.

The revised set-up will ensure that additional senior expertise is represented. The wider team's prior experience in developing and implementing similar programs lends credibility to their ability to successfully execute this project. The team's multi-disciplinary composition, comprising experts in education, psychology and public health, further strengthens the applicant's qualifications and enhances the project's potential.

3. Project feasibility

The proposed research has been designed with high feasibility of the proposed research plans due to several factors:

1. The study builds on an established intervention model.
2. The core elements of the physical literacy and mental health material already exist.
3. A school and a municipality have already been identified as project partners for the pilot study.
4. A thorough intervention logic model has been developed, which will allow for optional assessment of implementation and evaluation of outcomes.
5. The effectiveness trial: A power calculation has been undertaken which demonstrates that the proposed scale is well designed and powered to detect significant intervention effects.

6. All municipalities and now also nine schools have agreed to participate in the study once the funding has been secured.

Overall, the feasibility of the project appears to be reasonably addressed. The proposal resents concrete and achievable descriptions of the target population, recruitment strategies, and engagement of key stakeholders, including schools and teachers. The project team acknowledges potential challenges such as time constraints within the school curriculum and participant retention. The team and proposes tested and appropriate strategies to mitigate these challenges.

The inclusion of a pilot phase to test the program's feasibility is commendable and will help identify any necessary modifications before moving on to test the effectiveness of the intervention.

4. Probability by which the likely outcomes of the project could improve practice in the field

The likely outcomes of the 'My Life - I Decide' program may have the potential to significantly improve practice in the field of mental and physical activity literacy in schools. By incorporating evidence-based strategies and focusing on empowerment and decision-making, the program can equip Year 10 adolescents with the knowledge and skills necessary to make informed choices regarding their well-being, and develop a sense of autonomy for their own life using the life-psychological method.

This program has the potential to serve as a model for other schools and communities, thereby contributing to the broader adoption of effective mental and physical activity literacy interventions. This is especially the case given that the proposed study builds on the WHO health promoting schools (HPS) framework and takes into account that certain elements will require local/contextual adaptations.

5. Probability by which the likely outcomes could contribute to the research in the field

The 'My Life - I Decide' program offers opportunities for meaningful research contributions. The proposed evaluation plan, including both quantitative and qualitative measures, will generate valuable data to assess the program's impact and effectiveness.

The inclusion of process evaluation measures, such as fidelity assessments and participant feedback, will provide insights into program implementation, and further contribute to the existing body of knowledge in this very timely and rapidly evolving area.

Whilst not strictly necessary, consideration of longer-term follow-up assessments would strengthen the studies potential for generating valuable evidence.

6. Plan for dissemination

The proposal includes a simple and concise plan for dissemination, which demonstrates the applicant's commitment to sharing the project's findings. The intention to publish results in peer-reviewed journals and present at relevant conferences indicates a desire to reach both academic and practitioner audiences.

In addition, the proposal mentions the importance of engaging local education authorities and policymakers to facilitate wider adoption of the program, thus ensuring the research findings have a direct impact on policy and the lives of young people in Denmark.

7. Overall assessment of the project

<u>Background</u>
This is just a wider comment on the framing of the introduction. I understand that there is some literature that warrant educational approaches to increase health behaviors in young people. It would however be incredibly important to acknowledge that most of the literature presented will be affected by confounding.

Higher educational attainment and health literacy act as an index of higher socio-economic position, which is unequivocally linked to better health behaviors and health outcomes. It would be valuable to acknowledge this overarching issue.

Acknowledging this fact can also be leveraged to upsell the need for targeted health literacy programs to overcome this well-established SES-health gradient.

<u>State of the art</u>
The project builds on a very recent intervention evaluation led by the the Danish Center for Social Science Research (VIVE) in 2021, setting out a much-needed extension of the insights gained from this completed work.

Thank you for explicitly addressing the initial concerns regarding the reliance on education alone as the main applied behaviour change technique for this intervention. It is clearly explained how the program is linked to the HPS framework. The applicants explicitly acknowledge the importance of incorporating wider environmental (social/environmental) factors as vehicles to facilitate behaviour change.

<u>The My Life intervention</u>
Great use of participatory research methods nested within the development phase of the study. The program sounds like a thorough package, which warrants in-depth evaluation in the real world.

8. Other comments, including any proposals for improvement of the project.

Copyright and IP of the developed intervention materials: This project will include development of new and revised intervention materials which may be used for scaled-up delivery at the national level. I would suggest that legal advice and IP agreements are drafted and signed off by all contributing stakeholders in the early stages of the project to avoid conflict/uncertainty later on.

All mentioned elements of the project appear to be costed in the financial budget spreadsheet, but I am not able to comment on the value for money, as I am not familiar with salary and overhead costs in Denmark.

In conclusion, the 'My Life - I Decide' mental and physical activity literacy program demonstrates a strong project plan with a clear vision and objectives. The qualifications of the applicant and the collaborative nature of the research team enhance the project's potential for success.

The feasibility considerations, the potential to improve mental health of youth and to build on an existing partnership with the Vallensbaek municipality make for a unique and important project that is fundable.

Reiew 3

Ansøger: Charlotte D Klinker TrygFondens j.nr.: 158 775

		Fremra-gende	Meget god	God	Mindre god	Dårlig	Ved ikke
Projektets kvalitet	- Grundlæg-gende ide		x				
	- Originalitet		x				
	- Metodologi		x				
Ansøgerens kvalifikationer		x					
Gennemførligheden af projektet			x				
Sandsynligheden for at projektets forventelige resultater vil kunne forbedre praksis på området			x				
Sandsynligheden for at projektets forventelige resultater vil kunne bidrage til forskningen på området			x				
Formidlingsplan			x				
Samlet vurdering af ansøgningen			x				

Uddybning

1. Projektets kvalitet

På baggrund af de forbedringer, der er gennemført i den nye ansøgning, bedømmer jeg overordnet projektets kvalitet som meget tilfredsstillende. Ansøgerne har på tilfredsstillende vis svaret på de spørgsmål/anker, undertegnede havde til den første ansøgning. Som jeg opfatter det, er der også svaret tilstrækkeligt på de øvrige bedømmeres kommentarer.

Det efterfølgende er for det meste en gentagelse af mine kommentarer til den første ansøgning men med ændringer, der er baseret på den nye ansøgning.

Der er i ansøgningen lagt vægt på at bruge metoder, der i lignende projekter har vist positive resultater.

Det overordnede formål med My Life-projektet er at teste, om det kan anbefales til implementering på nationalt plan. Projektets mål er at øge 10. klasse-elevers trivsel, sundhedskompetencer, evne til at mestre udfordringer, opleve kontrol over begivenheder i eget liv (self-efficacy) og livsfærdigheder for at styrke elevernes forudsætninger for at gennemføre en ungdomsuddannelse.

My-Life interventionen er baseret på 1) den livspsykologiske metode (Bertelsen et al., 2020), som har påvist positive resultater på gymnasieelevers generelle greb om deres liv; og på 2) WHO Health Promoting School Approach (HPS), som har vist positive resultater på elevernes sociale og følelsesmæssige færdigheder (Goldberg et al., 2019).

Derudover skal følgende mål opfyldes:

1) Udvikle My Life-interventionen til at omfatte en uddannelses- og en skole- og lokalsamfundskomponent.

2) Gennemføre et pilotstudie på My Life-interventionen på én skole, og justere interventionen for at øge og sikre effektiviteten og accept og ejerskab blandt nøgleinteressenter.

3) Undersøge kontekst, mekanismer og implementeringsprocesser for My Life-interventionen på tværs af interventionsskoler.

4) Teste effektiviteten af My Life-interventionen i et kontrolleret ventelistedesign.

Projektets anden del skal bygge videre på pilotprojektet med intervention bestående af undervisning baseret på 'Bertelsens livspsykologiske metode', der er gennemført i Vallensbæk kommune. Dette projekt medførte, at eleverne i 10. klasse fik en øget viden om sammenhæng mellem fysisk og mental sundhed, ligesom eleverne oplevede et bedre klassemiljø.

Hidtidige interventioner på området har vist begrænsede eller ingen positive resultater. Derfor mener ansøgerne, at der skal findes strategier, som minimerer omkostninger og begrænser ekstraarbejdet for skolen og for underviserne. Det skal gøres gennem tre interventionskomponenter: (1) en pædagogisk uddannelseskomponent, (2) en skolekomponent og (3) en kommunal sundheds- og lokalsamfundskomponent.

Undervisningen i uddannelseskomponenten skal varetages af eksterne sundheds-konsulenter i samarbejde med lærerne. Sundhedskonsulenterne, der skal have erfaring med at formidle og undervise målgruppen, skal uddannes som 'ambassadører' i den livspsykologiske metode forud for implementeringen af uddannelsen 'livsfærdigheder og trivsel', som består af modulerne:

1) Fysisk sundhed (motion, kost, rygning, alkohol mv.) med vægt på fysisk aktivitet og kropsforståelse.

2) Mental sundhed (selvbevidsthed, evnen til at mestre livet og troen på egen formåen ud fra 'ti livsfærdigheder'.

Om dette skrev jeg sidst: "*I interventioner, der f.eks. har til formål at øge aktivitetsniveauet hos børn og unge, har det at bruge eksterne undervisere vist sig fordelagtigt i en implementeringsfase, og det har haft positiv indflydelse på resultaterne, men på længere sigt i et bæredygtighedsperspektiv, er det tvivlsomt, om det holder, da det jo er et ekstra led, der skal indføres i skolesystemet. I projektet hér, er det tilsyneladende ikke sundhedskonsulenter med en pædagogisk baggrund der skal være 'ambassadører', hvilket er overraskende, og der kan i høj grad stilles spørgsmål ved, om det på længere sigt er billigere at få eksterne undervisningskonsulenter ind i skolesystemet i stedet for uddannede lærere med interesse for og viden om området.*"

I den nye ansøgning er det bl.a. på baggrund af evalueringen af pilotprojektet begrundet, at de eksterne konsulenter har været værdifulde i implementeringen. I et længere perspektiv tror jeg stadig, det vil være svært at finde midlerne til sådanne ekstra ansatte, hvorfor interne 'ildsjæle/fyrtårne' skal findes.

For at udføre en feasibility-test vil forskellige forskningsværktøjer blive udviklet, pilottestet og justeret. En kombination af både kvalitative og kvantitative metoder vil blive anvendt til at give komplementær indsigt i fokusområderne. De vil blive udviklet, pilottestet og herefter justeret. Metoderne er blevet uddybet med bl.a. gode henvisninger til tilsvarende undersøgelser. Dette gælder også de statiske metoder.

2. Ansøgerens kvalifikationer

Ansøgningens kvalitet er absolut blevet højnet ved at gøre Charlotte Demant Klinker til hovedansøger. Men alle ansøgeres CV'er viser, at det er et kvalificeret ansøgergruppe, der alle har kvalifikationer og erfaringer inden for implementeringsforskning. Helle Stuart har stor erfaring i projektledelse i kommunalt regi, hvilket er helt centralt, når flere faggrupper og kommunale områder skal samarbejde.

3. Gennemførligheden af projektet

Baseret på de deltagende partneres kompetencer samt på tidligere gennemførte projekter af de projektansvarlige er det meget sandsynligt, at projektet kan gennemføres som planlagt.

4. Sandsynligheden for at projektets forventelige resultater vil kunne forbedre praksis på området

Det virker oplagt, at resultaterne fra projektet kan være med til at forbedre praksis på området, idet det er et velbeskrevet projekt, der arbejder med veludviklede metoder og inddrager alle centrale partnere på udmærket vis, herunder også eleverne. Om det også er bæredygtigt i et længere tidsperspektiv er uvist. Men det er heller ikke målet med projektet at vise dét.

5. Sandsynligheden for at projektets forventelige resultater vil kunne bidrage til forskningen på området

Det er et vigtigt område. Et velbeskrevet og velgennemført forskningsprojekt vil selvfølgelig bidrage til forskningen på området.

6. Formidlingsplan

Der er en velbeskrevet og tilfredsstillende formidlingsplan.

7. Samlet vurdering af ansøgningen

Samlet set er det en meget god ansøgning, der omhandler et problem, der bør være stort fokus på. Det er vigtigt at sikre, at børn og unge i denne aldersgruppe (specielt fra socialt udsatte områder) tilegner sig viden og færdigheder, der giver dem optimal baggrund for at kunne udnytte deres potentiale.

Det kan dog blive et stort problem, at der for nærværende er tanker om netop at nedlægge 10. klasserne. Men forhåbentlig vil et tilsvarende projekt, relateret til en skitseret ny 2-årig uddannelse for samme målgruppe, kunne gennemføres efter samme model.

8. Andre kommentarer, herunder forslag til forbedring af projektet

Ikke flere kommentarer.
